# Lysophosphatidylcholine Containing Anisic Acid Is Able to Stimulate Insulin Secretion Targeting G Protein Coupled Receptors

**DOI:** 10.3390/nu12041173

**Published:** 2020-04-22

**Authors:** Anna Drzazga, Marta Okulus, Magdalena Rychlicka, Łukasz Biegała, Anna Gliszczyńska, Edyta Gendaszewska-Darmach

**Affiliations:** 1 Institute of Molecular and Industrial Biotechnology, Faculty of Biotechnology and Food Sciences, Lodz University of Technology, Stefanowskiego 4/10, 90-924 Lodz, Poland; anna.drzazga@p.lodz.pl (A.D.); lukaszbiegala@o2.pl (Ł.B.); 2 Department of Chemistry, Wrocław University of Environmental and Life Sciences, Norwida 25, 50-375 Wrocław, Poland; marta.b.czarnecka@gmail.com (M.O.); rychlicka.magda@wp.pl (M.R.)

**Keywords:** phenolic acids, lysophosphatidylcholine, diabetes, insulin secretion, GPCR (G Protein Coupled Receptors)

## Abstract

Diabetes mellitus is a worldwide health problem with high rates of mortality and morbidity. Management of diabetes mellitus by dietary components is achievable especially at the initial stage of the disease. Several studies confirmed the antidiabetic activities of simple phenolic acids and lysophosphatidylcholine (LPC). The main goal of this study was to identify new potential insulin secretion modulators obtained by combining the structures of two natural compounds, namely *O*-methyl derivatives of phenolic acids and phospholipids. LPC and phosphatidylcholine bearing methoxylated aromatic carboxylic acids were tested as potential agents able to improve glucose-stimulated insulin secretion (GSIS) and intracellular calcium mobilization in MIN6 β pancreatic cell line. Our results show that LPC with covalently bonded molecule of *p*-anisic acid at the *sn*-1 position was able to induce GSIS and intracellular calcium flux. Notably, 1-anisoyl-2-hydroxy-*sn*-glycero-3-phosphocholine did not affect the viability of MIN6 cells, suggesting its potential safe use. Furthermore, we have shown that three G protein coupled receptors, namely GPR40, GPR55, and GPR119, are targeted by this LPC derivative.

## 1. Introduction

An increasing number of people suffering from diabetes is reflected in the growing market share of medications and nutraceuticals targeting this metabolic disorder. Diabetes mellitus (DM) is a dominant worldwide public health obstacle, affecting almost 463 million adults, a figure that by 2045 will rise to 700 million [1]. Given the complexity of the pathogenesis of DM, several treatment options have expanded. Besides insulin analogues, the approved pharmacologic treatment includes biguanides (metformin), which act on the liver to reduce gluconeogenesis and to cause a decrease in insulin resistance via increasing adenosine monophosphate-activated protein kinase (AMPK) signaling. While monotherapy with metformin is indicated for most patients at baseline, guidelines from the American Diabetes Association and the European Association for the Study of Diabetes for medical management of hyperglycemia recommend a second medication added to the treatment program, including sodium-glucose co-transporter-2 (SGLT2) inhibitors blocking the reabsorption of glucose in the kidney, incretin-based therapies such as glucagon-like peptide 1 (GLP-1) receptor agonists, dipeptidyl peptidase 4 (DPP-4) inhibitors stimulating insulin secretion and suppressing postprandial glucagon secretion in a glucose-dependent manner, and activators of the peroxisome proliferator-activated receptor-γ (PPARγ), namely thiazolidinediones which control normal skeletal muscle and hepatic insulin sensitivity and preserve pancreatic β-cell function. Other oral glucose-lowering medications such as sulfonylureas and meglitinide analogues acting directly on the islet β cells to close ATP-sensitive potassium channels and stimulate insulin secretion, acarbose, and miglitol, being α-glucosidase inhibitors which interfere with gut glucose production or amylin analogues suppressing glucagon release, are not commonly used. Furthermore, the guidelines emphasize the importance of individualizing the choice of medications, considering comorbidities, patient preferences, side effects, and cost [2,3,4].

Unfortunately, established monotherapies often becomes deficient in maintaining long-term glycemic control and are cognate to side effects [5]. Especially, several antidiabetic drugs may have important cardiovascular complications. In a class of thiazolidinediones, rosiglitazone was withdrawn from the market by the European Medicines Agency due to increased myocardial infarction risk. Rosiglitazone is also associated with weight gain, edema, and osteoporosis. Unlike rosiglitazone, another thiazolidinediones-containing drug, pioglitazone was reported to have a modest cardioprotective effect. However, treatment with pioglitazone may increase the risk of bladder cancer [6]. Although metformin does not have adverse cardiovascular effects, the most common are gastrointestinal, such as nausea, diarrhea, and/or abdominal discomfort. Metformin reduces intestinal absorption of vitamin B12 as well. A much rarer but more concerning adverse consequence of biguanide therapy is lactic acidosis [7]. Weight gain, hypoglycemia, and loss of efficacy represent the main problems related to meglitinides and sulfonylureas [2]. In addition, prolonged sulfonylurea’s use is associated with progressive deterioration in β cell function, whereas the most common side effects of GLP-1 analogues are vomiting, nausea, diarrhea, and injection-site reactions [8]. The most common side effects of DPP-4 inhibitors are headache, nasopharyngitis, nausea, and excessive touchiness [9]. The side effects of α-glucosidase inhibiting drugs include diarrhea, abdominal pain, and flatulence [10] while urogenital tract infections, reduction in bone formation, orthostatic hypotension, euglycaemic ketoacidosis, and the volume depletion are adverse effects of SGLT2 inhibitors [2]. Thus, new nutritional strategies to combat DM are necessary.

Dietary phytochemicals, including polyphenols, monoterpenes, terpenoids, stilbenes, lignans, coumarins, alkaloids, and others, have shown experimental or clinical activity for diabetes management and prevention [11]. In the family of polyphenols, phenolic acids, the secondary plant metabolites, are widely found in edible nuts, fruits, and vegetables, and 1–2 g/day of these constituents may be consumed in a human diet [12]. Dietary phenolic acids are known to play a significant role as health-promoting food ingredients [13]. Particularly, they stimulate insulin secretion, improve pancreatic β cell functionality, enhance glucose uptake, delay carbohydrate digestion, and inhibit protein glycation and insulin fibrillation [12,14]. One of the best-known properties of phenolic acids is the inhibition of α-glucosidase and α-amylase, important enzymes necessary during digestion of dietary polysaccharides [15]. However, phenolic acids are also able to reduce blood glucose by elevating plasma insulin levels [16]. There are limited reports of phenolic acids activating G protein coupled receptors (GPCR), but recently, Luna-Vital et al. have demonstrated that a phenolic-rich extract of red maize displayed the ability to handle obesity and diabetes by targeting GPR40 receptor. The extract contained phenolic acids (caffeic acid, hydroxycinnamic acid, vanillic acid, gallic acid, and protocatechuic acid), anthocyanins (cyanidin, peonidin-3-glucosides, and pelargonidin), and other flavonoids (luteolin and kaempferol). According to molecular docking, all of the phenolic acids identified as constituents of the extract showed potential interaction with GPR40 [17].

Unfortunately, phenolic acids and their derivatives, although commonly present in the diet, are characterized by low bioavailability and fast degradation rate that limits their biological effect [18]. New promising strategy to increase the stability of phenolic acids is their lipophilization with phospholipids and lysophospholipids [19,20,21]. We have already shown that O-methylated phenophosphatidylcholine (PC) and phenolysophosphatidylcholine (LPC) possess significantly increased anticancer properties [22,23]. LPC itself is a natural ligand of G protein-coupled receptors, which are expressed in insulin-producing β cells of pancreatic islets [24,25]. GPCRs have attracted the attention as potential pharmacological targets, as they regulate islet function and hormone secretions, ultimately controlling glucose homoeostasis, and have druggable binding site at the cell surface [26]. GPCRs form the largest human membrane protein family transducing signals from a variety of extracellular molecules to the intracellular milieu. After ligand stimulation, GPCRs undertake conformational shift and stimulate intracellular G proteins, initiating intracellular signaling. This activation involves the exchange of bound GDP for GTP by the G_α_ subunit of the G protein, leading to dissociation of the heterotrimeric protein complex into G_α_ and G_βγ_ subunits. Based upon the structure and downstream signaling cascade, the G_α_ subunit is mainly divided into 3 families: G_αs_, G_αi_, and G_αq_. GPCRs coupling to G_αs_ activate adenylate cyclase and cyclic AMP (cAMP) production. On the contrary, G_αi_ inhibits cAMP synthesis. GPCR coupling to G_αq_ triggers phospholipase Cβ and subsequently the formation of inositol 1,4,5-trisphosphate (IP_3_) and Ca^2+^ release from intracellular stores [27]. Although glucose levels are a primary regulator of insulin secretion, pancreatic β cells have numerous GPCRs that have complimentary or antagonistic actions on insulin secretion. In general, GPCRs that couple to G_αq_ and G_αs_ tend to stimulate insulin release by intracellular Ca^2+^ and cAMP production, respectively, while binding to G_αi_ inhibits insulin release by pancreatic β cells [28]. We have recently shown that LPC bearing oleoyl (18:1) and palmitoyl (16:0) fatty acid residue facilitate glucose stimulated insulin secretion (GSIS) from pancreatic cells. Those two LPC moieties recognize not only the previously discovered GPR119 receptor [29] but also GPR40 and GPR55 [24,30] which represent a totally novel antidiabetic approach. GPR40, activated mainly by medium- and long-chain free fatty acids (FFA) [31], is predominately expressed in insulin-secreting pancreatic β-cells and enteroendocrine L, K, and I-cells [32]. The clinical studies provided evidence that TAK-875, a GPR40 synthetic agonist, improved glucose control in type 2 diabetic patients in phase II clinical trials [33]. Our results indicate that GPR40 can be also activated by complex lipids like LPCs [24]. The receptor has been demonstrated to couple predominantly with G_αq_ family of G proteins [34]. Several research groups have confirmed that GPR40 plays a crucial role in stimulation of glucose-dependent insulin secretion from β-cells [31,34]. Therefore, development of GPR40 agonists is expected to be beneficial in DM treatment. As far as glucose homeostasis is concerned, LPC was also found to be one of endogenous agonists of GPR119 receptor, abundantly expressed in the intestinal tract, as well as α and β cells of pancreatic islets [28]. GPR119, defined sometimes as a fat sensor, is one of the most important and the best known GPCRs responsible for glucose-stimulated insulin secretion [35]. Activation of GPR119 is associated directly and indirectly with modulation of insulin secretion, as it stimulates insulin secretion from pancreatic β cells as well as GLP-1 and GIP from the intestinal L and K-cells, respectively [36,37,38]. GPR119 can be also activated by N-acylethanolamides, N-oleoyl-dopamine, and 2-oleoyl glycerol [35]. The main signaling pathway associated with GPR119 is related to activation of G_αs_; however, it is known that, dependent on the structure and amount of the agonist, the signaling pathway may shift towards other G proteins [36]. We have shown that phosphorothioate analogues of LPCs did not lead to significant cAMP increase but stimulated intracellular Ca^2+^ via GPR119-dependent manner [24]. GPR55 is another GPCR target for LPCs [30]. Both GPR119 and GPR55 receptors are also stimulated by endocannabinoids [14], but GPR55 is a well-documented target for lysophosphatidylinositol, which structurally resembles LPC [39]. The receptor is most often found coupled to G_αq_ [40] and is abundantly expressed in central nervous system, adrenal glands, testis, spleen, breast adipose tissues, and endothelium [39,41]. However, GPR55 has been also found expressed in pancreatic islets and gut enteroendocrine cells, implicating similar regulatory role towards pancreatic and intestinal hormone secretion as in the case of GPR119. Indeed, GPR55 is attracting more and more attention as a potential target for antidiabetic drug development, influencing secretion of insulin [42,43].

Since our previous studies clearly suggest that various acyl chains connected to glycerol backbone of the LPC structure as well as modification of the LPC backbone with methoxy group at the sn-2 position and hydrophilic phosphate head with a sulfur atom strongly influence biological effect both on the signaling (e.g., intracellular calcium mobilization) and secretory level (e.g., production of insulin) [24,25], we decided to study the antidiabetic activity of LPC bearing O-methyl derivatives of phenolcarboxylic acids. We decided to assess the potency of LPC analogues bearing natural methoxy derivatives of benzoic acids. This type of conjugate has not been tested so far in terms of insulin secretion modulation and related intracellular signaling with reference to the chosen GPCR targets (GPR40, GPR55, and GPR119). We synthesized the series of phosphatidylcholines and/or lysophosphatidylcholines containing the natural methoxy derivatives of benzoic acids: p-anisic acid (4-methoxybenzoic acid), m-anisic acid (3-methoxybenzoic acid), and veratric acid (3,4-dimethoxybenzoic acid) in the sn-1 and/or sn-2 positions of phosphatidylcholine. First, the phospholipid derivatives with p-anisic acid were studied. Biological evaluation was carried out by using the MIN6 cell line that has morphological characteristics of primary pancreatic β cells [44] and has confirmed expression of GPR40, GPR55, and GPR119 receptors [24]. In the next step, LPC with m-anisic and veratric acids were also examined as potential modulators of GSIS. We also investigated essential intracellular signaling, specifically intracellular calcium ([Ca^2+^]_i_) levels [45]. The most promising compound, 1-anisoyl-2-hydroxy-sn-glycero-3-phosphocholine, was selected for further study to determine the contribution of the three G protein coupled receptors in mediation of insulin secretion.

## 2. Materials and Methods

### 2.1. Chemicals and Reagents

Culture media and supplements, phosphate-buffered saline (PBS; pH 7.4), DMSO, and PrestoBlue Cell Viability Reagent were obtained from Life Technologies (Carlsbad, CA, USA). β-mercaptoethanol, ethanol, penicillin, neomycin, amphotericin B, and propidium iodide were purchased from Sigma-Aldrich (St. Louis, MO, USA). Bradford Protein Assay was obtained from *Bio*-*Rad* (Hercules, CA, USA), Lysis Buffer from R&D Systems, Inc. (Minneapolis, MN, USA), and Screen QuestTM Fluo-8 No Wash Calcium Assay Kit from AAT Bioquest, Inc. (Sunnyvale, CA, USA).

Specific antagonists of GPR40 (DC260126, depicted as DC) and GPR55 (CID16020046, depicted as CID) were obtained from Tocris Bioscience (Ellisville, MO, USA). The GPR119 antagonist (depicted as C8) was kindly provided by Pfizer (Groton, CT, USA) [46]. All antagonists were prepared as 10 mM stock solutions in DMSO and applied for cell culture studies at 2 μM working concentrations, as previously [24].

**Figure 1:**
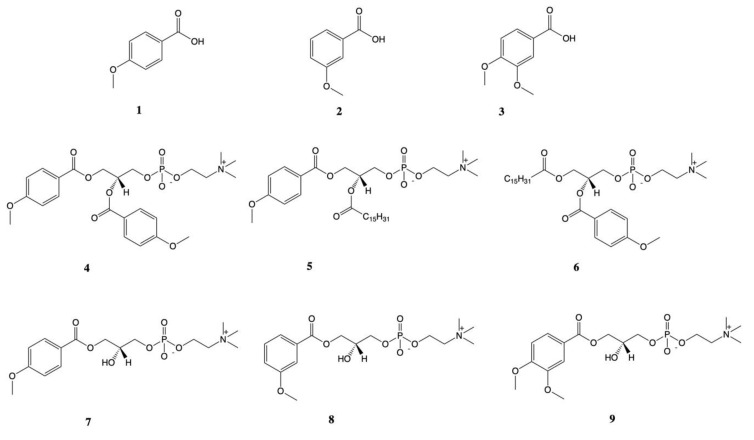
Studied methoxy derivatives of benzoic acid: *p*-anisic acid (**1**), 3-methoxybenzoic acid (**2**), veratric acid (**3**), and synthesized corresponding *O*-methylated phenophospholipids: 1,2-dianisoyl-*sn*-glycero-3-phosphocholine (**4**), 1-anisoyl-2-palmitoyl-*sn*-glycero-3-phosphocholine (**5**), 1-palmitoyl-2-anisoyl-*sn*-glycero-3-phosphocholine (**6**), 1-anisoyl-2-hydroxy-*sn*-glycero-3-phosphocholine (**7**), 1-(3-methoxy)benzoyl-2-hydroxy-*sn*-glycero-3-phosphocholine (**8**), and 1-veratroyl-2-hydroxy-*sn*-glycero-3-phosphocholine (**9**).

The enantimerically pure form of sn-glycero-3-phosphocholine (GPC) was purchased from Bachem (Bubendorf, Switzerland). p-Anisic acid (1), m-anisic acid (2), veratric acid (3) (Figure 1), dibutyltin (IV) oxide (DBTO), triethylamine (TEA), and oxalyl chloride were purchased from Sigma-Aldrich (Munich, Germany). Solvents for reaction, column chromatography, and thin-layer chromatography (TLC) were purchased from Merck (Darmstadt, Germany). NMR spectra were recorded on an Avance II 600 MHz spectrometer (Brüker, Billerica, MA, USA) working at a frequency of 600 MHz for ^1^H, 150 MHz for ^13^C and 243 MHz for ^31^P. Samples of all compounds were measured in a mixture of CDCl_3_:CD_3_OD (2:1, *v*/*v*). HRMS spectrum was recorded using the ESI technique on a spectrometer (ESI-Q-TOF Premier XE; Waters, Milford, MA, USA).

1,2-Dianisoyl-sn-glycero-3-phosphocholine (4), 1-anisoyl-2-palmitoyl-sn-glycero-3-phosphocholine (5), 1-palmitoyl-2-anisoyl-sn-glycero-3-phosphocholine (6), 1-anisoyl-2-hydroxy-sn-glycero-3-phosphocholine (7), and 1-veratroyl-2-hydroxy-sn-glycero-3-phosphocholine (9) (Figure 1) were originally synthesized at the Department of Chemistry, Wrocław University of Environmental and Life Sciences as described previously [22]. Solid compounds were solubilized in ethanol: DMSO solution (1:1) in 100 mM concentrations further diluted in PBS or cell culture media.

### 2.2. Synthesis of 1-(3-Methoxy)Benzoyl-2-Hydroxy-Sn-Glycero-3-Phosphocholine (8)

To a solution of 3-methoxybenzoic acid (0.257 g, 1.7 mmol) in anhydrous CH_2_Cl_2_ (10 mL) and catalytic amount (2 drops) of anhydrous, DMF was added oxalyl chloride (3 equiv, 1.23 mL, and 2.4 mmol) and the mixture was stirred at room temperature for 1 h. After this reaction time, the solvent and excess oxalyl chloride were subsequently removed in vacuum. The residual chloride was immediately used for the synthesis of 2-lysophosphatidylcholine.

Sn-Glycero-3-phosphocholine (0.257 g, 1 mmol) and DBTO (0.249 g, 1 mmol) were suspended in 12 mL of anhydrous propan-2-ol and refluxed. After a reaction time of about 1 h, the mixture was cooled to the room temperature and treated dropwise with TEA (0.242 g, 2.4 mmol) followed by chloride of methoxybenzoic acid (2.4 mmol). After stirring for 1 h, the reaction mixture was filtrated using diatomaceous earth (Celite^®^ 545, St. Louis, MO, USA) and the solvent was removed in vacuum. The crude product was purified by silica gel chromatography and analyzed by TLC according to previously reported procedure [22,23].

1-(3-methoxy) benzoyl-2-hydroxy-sn-glycero-3-phosphocholine, colourless greasy solid (46% yield, R_f_ 0.09); ^1^H NMR (600 MHz, CDCl_3_/CD_3_OD 2:1 (*v*/*v*)), δ: 2.92 (s, 9H, -N(CH_3_)_3_), 3.39 (m, 2H, CH_2_-β), 3.51 (s, 4H, –OCH_3_, –OH), 3.69 (m, 2H, CH_2_-3′), 3.83 (m, 1H, H-2′), 4.00-4.08 (two m, 4H, CH_2_-1′, CH_2_-α), 6.81 (m, 1H, H-4″), 7.04 (m, 1H, H-5″), 7.22 (m, 1H, H-2″), 7.30 (m, 1H, H-6″); ^13^C NMR (150 MHz, CDCl_3_/CD_3_OD 2:1 (*v*/*v*)) δ: 53.86 ((–N(CH_3_)_3_), 55.11 (–OCH_3_), 59.45 (C-α), 65.64 (C-1′), 66.16 (C-β), 66.86 (C-3′), 68.40 (C-2′), 114.59 (C-2″), 119.06 (C-4″), 121.79 (C-6″), 129.50 (C-5″), 130.94 (C-1″), 159.67 (C-3″), 166.68 (C-1); ^31^P NMR (243 MHz, CDCl_3_/CD_3_OD 2:1 (*v*/*v*)) δ: −3.95; HRMS (ESI): m/z calcd. for C_16_H_26_NO_8_P [M + H]^+^ 392.1474; found 392.1467. Data presented in the Supplementary Materials (Figures S1–S5).

### 2.3. MIN6 Cell Line Culture

The murine insulinoma MIN6 cells were kindly provided by Peter Bergsten (Uppsala University, Sweden) by permission of Jun-ichi Miyazaki (Division of Stem Cell Regulation Research, Osaka University, Japan) [44]. The applied culture conditions were standard for the cell line and provided as described previously [24]. The MIN6 cells were applied to experiments between passages 24–30.

### 2.4. Cell Viability

MIN6 cells seeded in 96-well plates in amount of 10^4^ cells per well 48 h prior to the experiment. Subsequently, the culture medium was changed to serum-free and supplemented with tested compounds. The ranges of tested compound concentrations and their respective solvent controls (EtOH/DMSO) were 5 μM, 10 μM, 25 μM, 50 μM, 100 μM, and 500 μM. Cell viability was quantified after 24 or 48 h of exposure to the tested compounds using PrestoBlue Cell Viability Reagent according to the manufacturer’s instructions by measuring the fluorescent signal at F530/590 nm. The obtained fluorescence values were used to calculate cell viability expressed as the percentage of the viability of the untreated control cells (cells treated with equal volume of the vehicle instead of the preparation).

### 2.5. Glucose-Stimulated Insulin Secretion (GSIS)

MIN6 cells were seeded on 24-well plates in amount of 2 × 10^5^ per well 48 h prior to the experiment. Confluent cells were initially pre-incubated for 60 min with a pH 7.4 calcium buffer pH 7.4 as previously [24], supplemented with 2 mM glucose. Subsequently, cells were incubated in the same buffers with tested compounds at either 25 or 50 µM working concentrations and/or GPCR antagonists (2 µM of DC, CID, and C8) for 30 min. The buffer samples were collected, and the same cells were incubated for another 30 min with fresh buffer supplemented with 20 mM glucose and respective test compounds. After collection of buffer samples from the high glucose conditions, cells were washed with cold PBS and lysed with 0.1 M HCl. Both the buffer samples and cell lysates were stored below −20 °C for further analysis. Buffer samples were used for insulin secretion measurements by competitive ELISA, as previously described [24]. Quantities of secreted insulin were normalized to protein contents in respective cell lysates measured according to Bradford Protein Assay.

### 2.6. Calcium Flux Measurements

MIN6 cells were seeded onto 96-well plates in amounts of 4 × 10^4^ cells per well 24 h prior to the experiment. The intracellular calcium concentration [Ca^2+^]_i_ was assessed with the Screen QuestTM Fluo-8 No Wash Calcium Assay Kit according to the supplier’s protocol. Prior to experiment, MIN6 culture medium was substituted with the buffer supplemented with either 2 mM or 20 mM glucose. Just before the assay, propidium iodide (PI) was added at a final concentration of 1 µg/mL to monitor possible membrane permeabilization caused by the investigated compounds [47]. Real-time [Ca^2+^]_i_ mobilization was measured after stimulation with chosen compound and/or GPCR antagonists (applied at the same working concentrations as in the case of GSIS experiments). Calcium flux and PI intercalation were monitored simultaneously by the change in fluorescence (excitation/emission = 490/520 nm and 535/617 nm, respectively) following stimulation and corrected for background fluorescence. The obtained fluorescence reads were referred to the results obtained after stimulation of the cells with the compound solvent.

### 2.7. Statistical Analysis

Results are presented as means of 2–6 repeated experiments (3–4 biological repeats each) ± SEM, and groups of data were compared using one-way ANOVA with Bonferroni post hoc test. *p* < 0.05 was regarded as statistically significant. The comparisons were performed between results obtained after stimulation of the cell model with investigated compounds versus respective solvent control (EtOH/DMSO). The analysis was performed separately for high glucose conditions (*) and for low glucose conditions (.). Whenever the GPCR-mediated activity was tested, the statistically significant difference was depicted between the cell model treated with the compound of interest and the cell model treated with the compound of interest and a specific receptor antagonist simultaneously (^#^). Statistical significance of the obtained results was determined with GraphPad Prism v. 8.3 (GraphPad Software, La Jolla, CA, USA).

## 3. Results

### 3.1. Synthesis of Phospholipids Containing Methoxy Derivatives of Benzoic Acid

The series of enantiomerically pure phosphatidylcholines and lysophosphatidylcholines with natural R-configuration of chiral center containing selected methoxy derivatives of benzoic acid at the sn-1 and/or sn-2 positions were synthesized as the potential antidiabetic agents (Figure 1).

Among this group of natural acids, we focused our attention on the p-anisic acid and its derivatives, trying to determine the relationship between the position of methoxy group in the aromatic ring and biological effect of synthesized phospholipids (PLs) as the GPR40, GPR55, and GPR119 agonists. p-Anisic, m-anisic, and 3,4-dimethoxybenzoic acids were selectively incorporated into the structure of PLs according to the procedure described earlier [10]. Products were obtained in good yields (28–66%) and their structures and purities were confirmed by NMR spectroscopy and HPLC chromatography, respectively.

### 3.2. The Influence of Conjugates of Phosphatidylcholine and Lysophosphatidylcholine with p-Anisic Acid on MIN6 Viability

**Figure 2:**
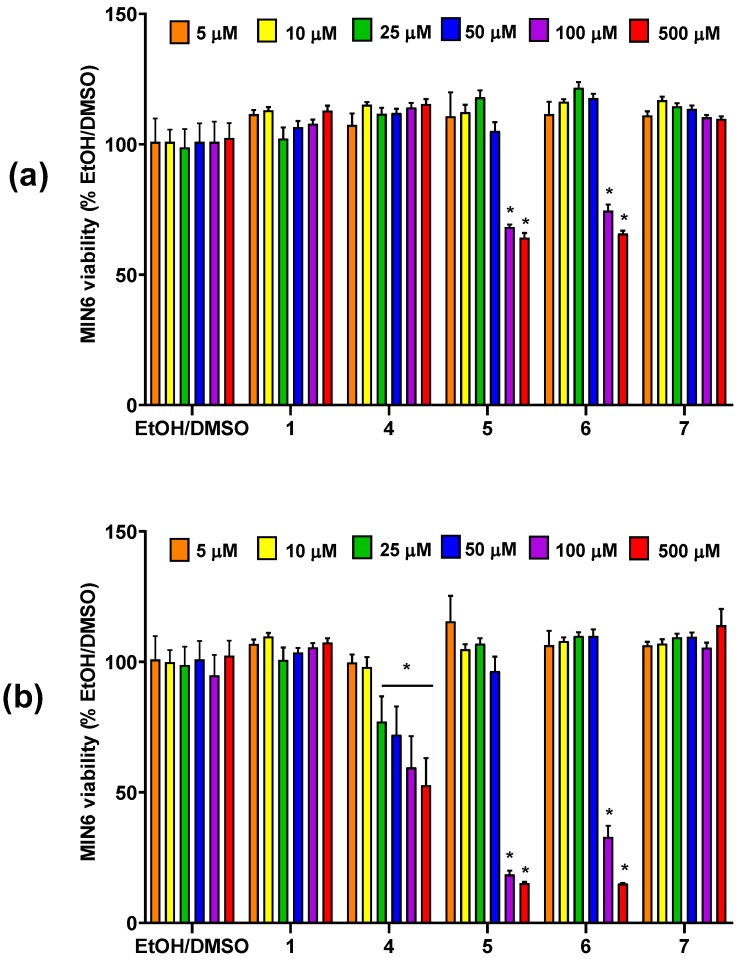
MIN6 cells viability after 24-h (**a**) and 48-h (**b**) treatment in fasting conditions with *p*-anisic acid (1), phosphatidylcholines substituted with at anisic and/or palmitoyl acyl residues in different configurations (4–6), and 1-anisoyl-lysophosphatidylcholine (LPC) (7) in the range of 5–500 μM concentrations. The viability is expressed as % of viable cells after treatment with respective quantities of the compound solvent (EtOH/DMSO). * *p* < 0.05 is regarded as significantly different from EtOH/DMSO.

Potential cytotoxicity of conjugates of phosphatidylcholine with p-anisic acid in MIN6 cell line was evaluated in the range of 5–500 μM concentrations with resazurin-based PrestoBlue Cell Viability Reagent (Figure 2). Experiments were performed at fasting conditions that allow recognition of the highest compound concentration with a neutral effect on cell growth when existing in culture medium without any supplemental substances of masking qualities. The potential background fluorescence of tested compounds incubated solely with culture media showed no statistically significant differences as compared to control media (data not shown).

In most cases, no cytotoxic effect was noticed after 24 h of incubation. Only heterosubstituted phosphatidylcholines 1-palmitoyl-2-anisoyl-sn-glycero-3-phosphocholine (6) and 1-anisoyl-2-palmitoyl-sn-glycero-3-phosphocholine (5) reduced cell survival by approx. 20–40% at the two highest concentrations (100 μM and 500 μM) used. Their cytotoxic effect deepened over time, which resulted in a very significant reduction in the survival of MIN6 cells up to just ca. 15% of control cell culture after incubation with the above derivatives used at a concentration of 500 μM. It is noteworthy that concentrations of these two PCs lower than 100 μM did not affect the cellular growth (Figure 2a). Moreover, after 48 h, homosubstituted 1,2-dianisoyl-sn-glycero-3-phosphoholine containing p-anisic acid in both sn-1 and sn-2 positions (4) also proved to be cytotoxic. The toxic effect of this compound was observed even at 25 μM, which resulted in a depletion in cell survival by half at the highest concentration tested. After two days of stimulation of MIN6 cells, no decrease in their survival was observed under the influence of 1-anisoyl-2-hydroxy-sn-glycero-3-phosphoholine (7) and anisic acid (Figure 2b).

### 3.3. The Influence of Conjugates of Phosphatidylcholine and Lysophosphatidylcholine with p-Anisic Acid on GSIS and Intracellular Ca^2+^ Mobilization in MIN6 cells

**Figure 3:**
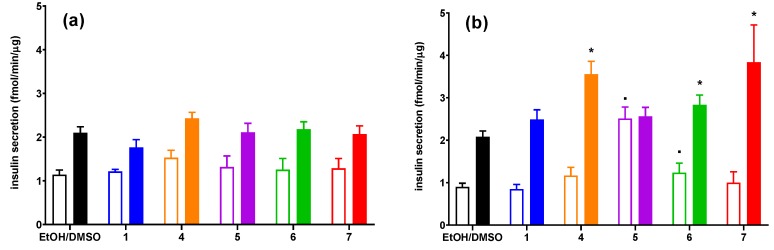
Glucose-stimulated insulin secretion (GSIS) in MIN6 cells stimulated with *p*-anisic acid (1), phosphatidylcholines substituted with at anisic and/or palmitoyl acyl residues in different configurations (4–6), and 1-anisoyl-LPC (7) at either 25 μM (**a**) or 50 μM (**b**) concentration versus compound solvent control (EtOH/DMSO). Results are presented for insulin secreted at 2 mM glucose conditions (open bars) and 20 mM glucose conditions (closed bars). *p* < 0.05 for secretion significantly different from 2 mM glucose control (.) and 20 mM glucose control (*).

Insulinotropic activities of conjugates of phosphatidylcholine with p-anisic acid were examined in 2 mM and 20 mM glucose concentration in the MIN6 cell line. Taking into account cytotoxic properties of chosen compounds, concentrations of 25 μM (Figure 3a) and 50 μM (Figure 3b) were chosen for GSIS studies. High glucose concentration itself caused ca. 2–2.5-fold increase of secreted insulin, in agreement with other studies [48].

None of the analyzed compounds used at the lower dose (25 μM) had a statistically significant effect on glucose-stimulated insulin secretion (Figure 3a). Among compounds tested at a concentration of 50 μM, compounds 5 and 6 caused significant unfavorable grow in insulin secretion at low glucose concentration. In addition, anisic acid alone did not affect insulin secretion under both low and high glucose concentrations. At the same time, 4, 6, and 7 augmented GSIS at 20 mM glucose. 6 caused the weakest reaction (ca. 1.5-fold increase in GSIS), whereas 4 and 7 exerted 2-fold rise in insulin secretion compared to control conditions (Figure 3b).

An increase in [Ca^2+^]_i_ is principal for insulin release in pancreatic cells [45]. Thus, changes in the concentration of intracellular calcium ions under the influence of the tested compounds were examined based on the fluorescence intensity of the Fluo-8 calcium probe. Intracellular calcium mobilization was monitored in MIN6 cells in a period of 3 min after stimulation with 25 μM and 50 μM concentrations of conjugates of phosphatidylcholine and lysophosphatidylcholine with p-anisic acid in a buffer containing 2 mM or 20 mM glucose. To monitor cell membrane permeability during exposure to tested compound, propidium iodide staining was co-monitored with calcium flux [47].

**Figure 4:**
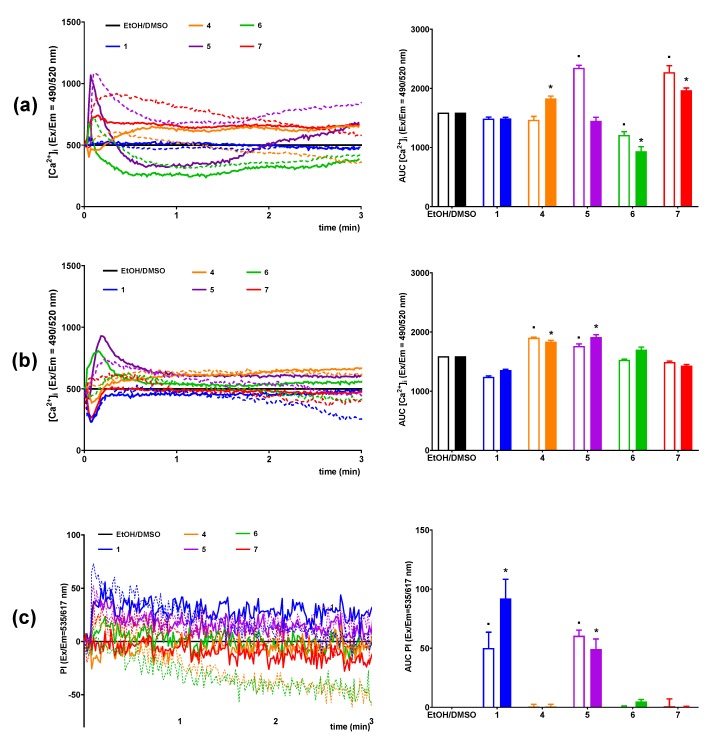
Intracellular Ca^2+^ mobilization in MIN6 cells stimulated with *p*-anisic acid (1), phosphatidylcholines substituted with at anisic and/or palmitoyl acyl residues in different configurations (4–6), 1-anisoyl-LPC (7), or compound solvent (EtOH/DMSO) at 20 mM (**a**) and 2 mM glucose conditions (**b**), with simultaneous monitoring of membrane integrity via PI incorporaton (**c**). The results are presented as the real-time kinetics of [Ca^2+^]_i_ and [PI] changes inside the cell during 3-min monitoring as well as AUC. The compounds were tested in 25 and 50 μM concentrations indicated as dashed or full lines and open or closed bars respectively. *p* < 0.05 for secretion significantly different from 2 mM glucose control (.) and 20 mM glucose control (*).

The calculated increase of AUC (area under the curve) parameter value of the calcium responses was statistically significant in the case of 4 and 7 (Figure 4a); however, the kinetics of [Ca^2+^]_i_ flux varied depending on the structure of the derivative studied. The initial increase in intracellular calcium mobilization under the influence of 7 remained stable throughout the whole monitoring period. Four did not contribute to the rapid growth of [Ca^2+^]_i_ just after stimulation of MIN6 cells, like in the case of 7, but further caused a slow but stable mobilization of Ca^2+^ ions (Figure 4a). The increase in AUC, used to estimate the total amount of calcium mobilized, was not observed for *5, 6*, and 1. Five and 6 induced only a short-term mobilization of [Ca^2+^]_i_ immediately after cell stimulation and then caused a sharp decrease in [Ca^2+^]_i_. During incubation of MIN6, cells under conditions of low glucose concentration 6, 7, and 1 did not demonstrated significant effect on the mobilization of [Ca^2+^]_i_ (Figure 4b). Importantly, simultaneous propidium iodide staining revealed that 5 and 1 caused significant membrane perforation (Figure 4c).

### 3.4. The Influence of Conjugates of Lysophosphatidylcholine with Veratric and 3-Methoxybenzoic Acid on Viability, GSIS and Intracellular Ca^2+^ Mobilization in MIN6 Cells

**Figure 5:**
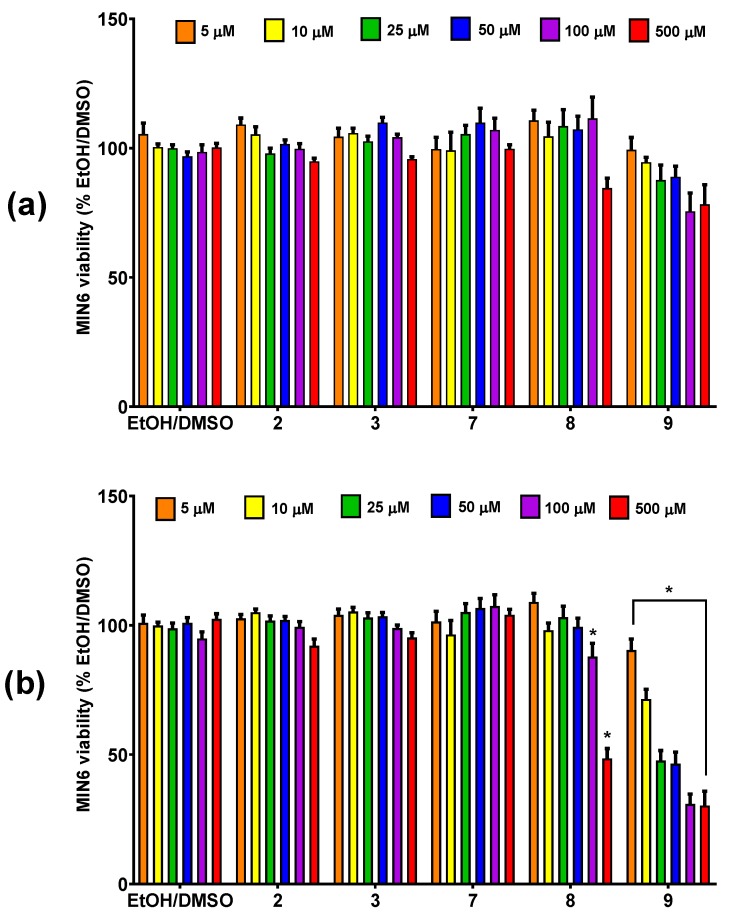
MIN6 cells viability after 24-h (**a**) and 48-h (**b**) treatment in fasting conditions with 3-methoxybenzoic acid (2), veratric acid (3), and LPCs (7–9) in the range of 5–500 μM concentrations. The viability is expressed as % of viable cells after treatment with respective quantities of the compound solvent (EtOH/DMSO). * *p* < 0.05 is regarded as significantly different from EtOH/DMSO.

Summing up the results of the ability of PC and LPC derivatives conjugated with p-anisic acid to release insulin by MIN6 cells, the most preferred GSIS activator turned out to be lysophosphatidylcholine containing anisic acid in the sn-1 position. Additionally, this compound stimulated intracellular calcium mobilization under conditions of high glucose concentration and, at the same time, showed no cytotoxicity. Therefore, we decided to assess the activity of other LPC derivatives of benzoic acid, which possess methoxy groups in a different position of aromatic ring than in p-anisic acid. Eight and 9 containing 3-methoxybenzoic acid and 3,4-dimethoxybenzoic acid, respectively, were under study. Veratric acid and 3-methoxybenzoic acid alone served as control acids. Both LPCs exhibited higher cytotoxic effect against MIN6 cells than 7, whereas free form of these acids did not inhibit cell viability. The most significant decrease in survival of MIN6 cells was observed after treatment with 9 reducing cell survival from ca. 80% up to ca. 20% at concentrations of 5 μM and 500 μM, respectively, after 48 h of incubation. Eight was also toxic, however at higher concentrations applied (Figure 5).

**Figure 6:**
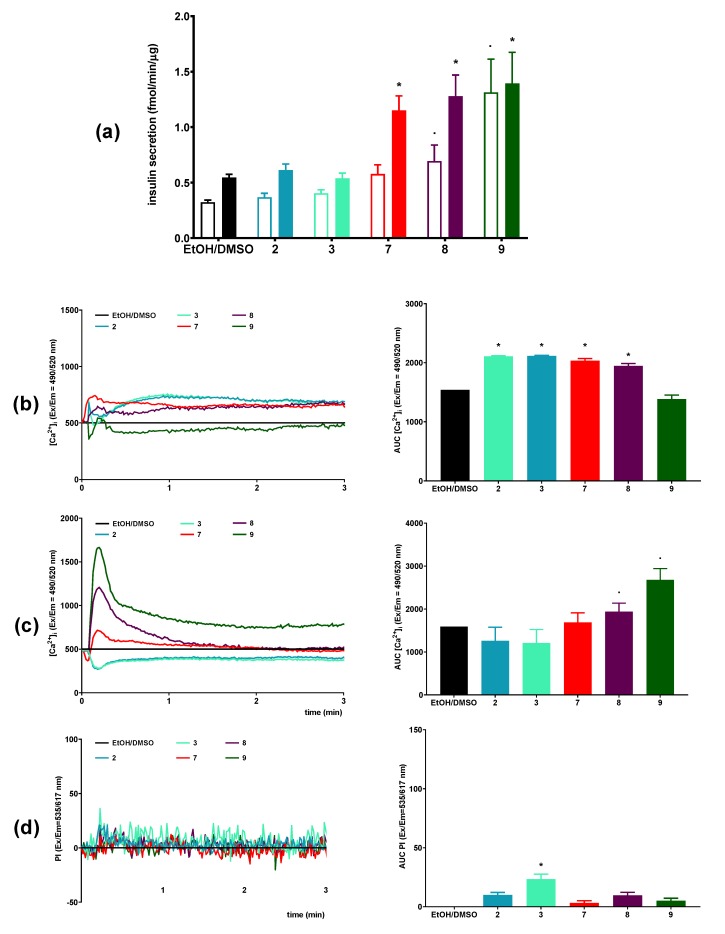
Influence of 3-methoxybenzoic acid (2), veratric acid (3), and LPCs (7–9) at 50 μM concentration on MIN6 cells versus compound solvent control (EtOH/DMSO) in terms of GSIS (**a**) and [Ca^2+^]_i_ mobilization at 20 mM (**b**) and 2 mM (**c**) glucose conditions with simultaneous monitoring of membrane integrity ((d) presented for 20 mM glucose conditions). For GSIS (**a**), results are presented for insulin secreted at 2 mM glucose conditions (open bars) and 20 mM glucose conditions (closed bars). For [Ca^2+^]_i_ mobilization (**b**,**c**) and membrane integrity (**d**), the results are presented as the real-time kinetics of [Ca^2+^]_i_ and [PI] changes inside the cell during 3-min monitoring as well as AUC. *p* < 0.05 for secretion, AUC [Ca^2+^]_I_, or AUC PI significantly different from 2 mM glucose control (.) and 20 mM glucose control (*).

Eight and 9 appeared to be the potent GSIS stimulators demonstrating ca. 2.5-fold higher efficiency compared to control sample. Unfortunately, those compounds increased the amount of secreted insulin also in low glucose concentrations up to 4-fold in the case of 9 (Figure 6a). Under 2-mM glucose concentration, 8 did not induce mobilization of intracellular calcium flux in contrast to 9 which caused a very rapid increase in [Ca^2+^]_I_ (Figure 6b). Eight also stimulated the mobilization of [Ca^2+^]_i_ in the presence of 20 mM glucose, whereas 9 was inactive (Figure 6C).

### 3.5. The Role of GPR40, GPR55, and GPR119 in GSIS and Intracellular Ca^2+^ Mobilization Induced by 1-Anisoyl-2-Hydroxy-Sn-Glycero-3-Phosphocholine

Among all phospholipids containing methoxy derivatives of benzoic acid studied, hereby only 7 was not toxic in the whole range of doses and, simultaneously, it increased insulin secretion under hyperglycemic condition without enhancing insulin release at low glucose level. Its favorable properties encouraged us to carry on further study to better elucidate the mechanism of action. Therefore, 7 was selected to evaluate the share of the three G protein coupled receptors crucial in insulin secretion. Selective antagonists (DC, CID, and C8 of GPR40, GPR55, and GPR119, respectively) were applied to assess insulin secretion and intracellular Ca^2+^ mobilization evoked by 7 used at 50 μM concentration.

**Figure 7:**
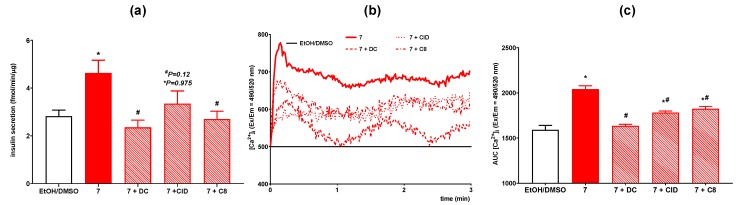
Insulin secretion (**a**) and intracellular Ca^2+^ mobilization ((**b**) kinetic read, (**c**) AUC) in MIN6 cells at 20 mM glucose conditions stimulated with **7** against GPR40, GPR55, and GPR119 antagonists (depicted as DC, CID, and C8 respectively). *p* < 0.05 for insulin secretion and [Ca^2+^]_i_ mobilization significantly different from EtOH/DMSO control (*) and from MIN6 cells untreated with receptor antagonists (^#^).

Insulinotropic activity of 7 in the presence of each antagonist tested was significantly reduced compared to MIN6 cells stimulated with compound alone. As a result, GPR40 and GPR119 antagonists completely abolished the secretion of insulin enhanced by the presence of 7. Application of GPR55 antagonist decreased insulin release stimulated with 7 by ca. 25% (Figure 7a).

In accordance with GSIS, mobilization of [Ca^2+^]_i_ stimulated by 7 was also abolished in the presence of each of the GPCR receptors’ antagonists. Blocking of GPR40 led to decrease of AUC parameter value to the level of unstimulated cells. In the case of GPR55 and GPR119 antagonists, stimulatory effect evoked by 7 was reduced by ca. 13% and ca. 11%, respectively (Figure 7b,c).

## 4. Discussion

Our data demonstrate that LPCs bearing natural methoxy derivatives of benzoic acids may act as new potential insulin secretion modulators targeting GPR40, GPR55, and GPR119 receptors. Phenolic acids alone have been already proved to possess antidiabetic activity [12,14]; however, they undergo rapid metabolism after oral administration in humans [18]. Their therapeutic efficacy is also limited by their low oral bioavailability and solubility. Since bioavailable products should be characterized by the balance between hydrophilicity and lipophilicity to dissolve in the biological fluids and to cross cell membranes, respectively, one of the methods increasing the biological activity of these compounds in vivo is their application in lipid matrix. The beneficial role of phospholipids in enhancing the therapeutic efficacy of some molecules having poor oral absorption have been demonstrated. Example in this aspect may be silybin—the main and the most active flavonoid from complex silymarin, an extract isolated from *Silybum marianum* L., which is known as a powerful carrier protecting human organism against the disfunction of liver and gall bladder and naturally occurring toxins (e.g., snake bites, mushroom poisoning, and insect stings) [49,50]. It was proven that the bioavailability of silybin increases five times during oral administration when it is administered in the form of complex with phosphatidylcholine (Siliphos^®^, Indena, WA, USA) [51]. Also, curcumin–phospholipid complex persisted for a longer period of time in rats with a higher relative bioavailability and maintained effective concentration of curcumin for a longer period of time [52]. In addition, other studies suggested that mixtures of phenolic compounds with phospholipids advanced physiological and biological properties compared to unmodified correspondents [53]. However, such complexes are rather unstable, and therefore, it is logical to surmise that covalent linkages of PLs with phenolic acids will have additive characteristics (emulsifying and antioxidant) or even synergistic, which may boost their application. For these reasons, in recent years, many attempts have been made to modify physiochemical properties and chemical structure of phenolic acids in order to break mentioned restrictions.

Many attempts have been made to develop the chemical and enzymatic methods of lipophilization of phenolic acids with fatty alcohols and acylglycerols [54,55] Currently, special attention is paid to production of conjugates of polyphenols with PLs where active molecule is attached to glycerol backbone of PC [19,20,56,57]. Phospholipid conjugates prepared according to this strategy can form liposomes or can enhance the incorporation of active molecule into PLs-based delivery systems [58]. Barriers such as the first-pass effect and other metabolic factors limiting the bioavailability and effectiveness of phenolic compounds can be removed in this way. These molecules can avoid transfer to the liver via the portal vein, conversion into sulfonic and glucuronide derivatives, and subsequent excretion via urine [59]. It is known that, after lipophilization, active molecules/drugs can exhibit increased interactions with cell membranes [60]. It was also proven that, after conjugation of active molecules/drugs with lipids, formed hybrids are transported via lymphatic system, which functions in the transport of dietary lipids to lymphatic capillaries [61,62]. Porters’s group reported that mycophenolic acid triglyceride conjugate showed significant enhancement in lymphatic drug transport and demonstrated a great potential for lymphatic targeting [63,64]. We have already shown that lipophilization of methoxy derivatives of benzoic and cinnamic acids significantly increased their anticancer properties in comparison to the free form of these acids [22,23].

However, it should be noted that natural phospholipids themselves have also limited therapeutic utility due to short half-life [65]. Orally administrated phospholipids are first hydrolyzed by phospholipase A_2_ (PLA_2_) in the intestinal lumen to produce the corresponding lysoderivatives, which, after absorption by the enterocyte, are reacetylated prior to becoming involved with stabilization of the surface of lipoproteins [61]. However, the process of hydrolysis of modified PLs is significantly slower and depends on the position of conjugation of active molecule with the skeleton of PLs. Active moiety can be attached to the glyceride backbone, replacing the sn-1 and/or sn-2 positioned fatty acids. If the conjugation is in the sn-1 position, only hydrolysis of the fatty acids from the sn-2 position catalyzed by phospholipase A_2_ takes place. PL-conjugates with direct conjugation between the active moiety and the phospholipid produce a complex having unique properties. For the phosphatidylcholine with attached valproic acid directly to the sn-2 position, it was confirmed that complex is stable and does not undergo degradation in the gut lumen. This hybrid permeates through the gut wall, enters intact to the enterocyte, associates to the chylomicron, and reaches the systemic blood circulation via the lymphatic route [66]. When the active molecule is attached to the sn-2 position via linker, it was confirmed for a series of PL-diclofenac conjugates that 6-carbon linker is the optimal length with the greatest extent of activation by PLA_2._ Hybrids with shorter linker were not hydrolyzed by PLA_2_ [67].

Taking the above into account, we focused our attention on the phospholipid conjugates containing natural methoxy derivatives of benzoic acid with well-documented pro-health properties useful in prevention of several chronic diseases. These methoxy derivatives have been reported to have hepatoprotective, antibacterial, anti-inflammatory, and antioxidative activities [68,69,70,71]. However, their antidiabetic effects have not been investigated so far. To determine nontoxic doses for further studies, in the viability studies, conjugates of PCs and PLPs with p-anisic acid, m-anisic acid, and veratric acid were tested in MIN6 pancreatic cells exposed for 24 and 48 h to drugs at six concentrations ranging from 5 to 500 µM. Such concentrations range was chosen based on our previous observations. IC_50_ values for phosphocholines containing anisic/veratric acid evaluated towards cancer cell lines, namely human breast (MCF-7), leukaemia (MV4-11), liver (HepG2), lung (A549), and colon (LoVo) cancer, ranged between 16.7 and 463.7 μM. On the other hand, in our previous study devoted to methoxy and phosphorothioate analogues of LPCs, it was revealed that, in the case of 10-µM concentration of natural and modified LPC species, there was no significant effect on viability of the β cell model yet 25 µM appeared to be toxic [72]. We have also selected long terms (24 h and 48 h incubation time) with the investigated compounds since, previously, we have checked short (4 h of incubation) time points which revealed that all of the tested LPCs, both at 10 µM and even at 25 µM concentrations, did not significantly inhibit pancreatic cell proliferation [24,72]. Since in theory, it is possible that, at 24 h, the cells are not showing complete or full influence of the treatment, we have additionally evaluated viability at 48 h to see more pronounced effect.

LPC analogues studied hereby appeared to be less toxic as compared to unmodified LPCs. LPCs are cone-shaped with hydrophobic tails and polar heads and can efficiently be inserted into lipid bilayers disrupting plasma membrane integrity. Application of LPCs in amounts close to critical-micelle concentration (CMC) may lead to rupture of the lipid bilayer or even its total solubilization [73]. CMC for LPC depends on the length and saturation of the fatty acid constituting the hydrophobic part and for 16:0 LPC is about 7–10 µM [74,75]. LPC has been also shown to injure mouse aorta endothelial cells by inducing membrane perforation and cell lysis [76]. In PC and LPC studied, hereby in most cases, no cytotoxic effect was observed after 24 h of incubation. After 48 h, the toxicity continuously increased but most of the toxic effects were limited to higher concentrations (100 and 500 µM). After 48 h, toxicity was indicated in the order of 5–6 > 9 > 4–8 > 7 at concentration of 500 µM. Taking into account LPC derivatives, a significant difference in their toxicity is clearly evident with the number and position of methoxy groups in the benzoic acid. In the case of compounds containing anisic acids, LPC with p-anisic acid did not affect cell viability even at 500 µM while LPC with m-anisic contributed to 50% decrease of cell growth. LPC with veratric acid appeared to be the most toxic. In our previous studies, 9 also negatively affected the survival of many other cell types including MV4-11, MCF-7, and LoVo cancer cell lines [22]. It can be suggested that a bulky, disubstituted benzene ring in 9 could be responsible for the toxic effect.

We have proved that LPC bearing natural methoxy derivatives of benzoic acids may act as new potential insulin secretion modulators. During glucose-stimulated insulin secretion, we have employed conditions that are used in most GSIS protocols. Cells are routinely exposed to low glucose prior to a glucose challenge independently if experiments are conducted with pancreatic cell cultures [77], islets isolated after the perfusion, and digestion of the pancreas with collagenase or during oral glucose tolerance test (OGTT) in vivo [78]. In humans, patients consume the correct amount of glucose (established by weight, up to 75 g). Plasma insulin peak occurs at 30 min during normal glucose response [79], and such time point was used in our studies.

One of the most important results of this study is demonstrating that 7 contributes to insulin secretion via three GPCRs, namely GPR40, GPR55, and GPR119 involved in maintaining lipid and carbohydrate homeostasis. To confirm this observation, we monitored downstream basic intracellular signaling, namely intracellular calcium levels, which plays a major role in augmenting GSIS. Intracellular calcium mobilization along with stimulation of insulin secretion evoked by 7 was observed only at stimulatory high glucose concentration. This phenomenon is extremely important because the positive effect on insulin secretion must be relevant physiologically. Besides, raised basal insulin secretion under fasting conditions along with deficient stimulated insulin secretion is an important indication of type 2 diabetes [80]. Furthermore, 7 did not affect the viability of MIN6 cells up to the highest concentration of 500 µM, suggesting its potential safe use. This contrasts starkly with the previously published results showing high cytotoxicity observed for natural LPCs since our studies revealed that only 10 µM concentration of natural LPC species is not toxic [73]. The finding that 7 acts as an agonist of GPR40, GPR55, and GPR119 offers a new therapeutic approach in the therapy of DM because of its potential beneficial effect beyond improvement of insulin secretion. However, further studies are needed to confirm this observation under in vivo conditions to assess its stability and bioavailability.

## Supplementary Materials

The following are available online at https://www.mdpi.com/2072-6643/12/4/1173/s1, Figure S1: ^1^H NMR spectrum, Figure S2: ^13^C NMR spectrum, Figure S3: ^31^P NMR spectrum, Figure S4: ^1^H–^1^H COSY spectrum, Figure S5: HSQC spectrum.
