# Supplementary material for: Lysophosphatidylcholine Containing Anisic Acid Is Able to Stimulate Insulin Secretion Targeting G Protein Coupled Receptors
Source: Nutrients. 2020 Apr 22;12(4):1173. doi: 10.3390/nu12041173 (PMC7230207; doi:10.3390/nu12041173)
Supplement: Supplementary file 1 [file nutrients-12-01173-s001.pdf]

nutrients

Supplementary Materials

Lysophosphatidylcholine Containing Anisic Acid is able to Stimulate Insulin Secretion Targeting G Protein Coupled Receptors

Anna Drzazga [1], Marta Okulus [2], Magdalena Rychlicka [2], Łukasz Biegała [1], Anna Gliszczyńska [2,*], Edyta Gendaszewska-Darmach [1,*]

[1] Institute of Molecular and Industrial Biotechnology, Faculty of Biotechnology and Food Sciences, Lodz University of Technology, Stefanowskiego 4/10, 90-924 Lodz, Poland; anna.drzazga@p.lodz.pl (A.D.); lukaszbiegala@o2.pl (Ł.B.)

[2] Department of Chemistry, Wrocław University of Environmental and Life Sciences, Norwida 25, 50-375 Wrocław, Poland; marta.b.czarnecka@gmail.com (M.O.); rychlicka.magda@wp.pl (M.R.)

* Correspondence: anna.gliszczynska@wp.pl (A.G.); Tel.: +48 713205183; edyta.gendaszewska-darmach@p.lodz.pl (E.G.–D.); Tel.: +48-426313443

Content

Figure S1: ^{1}H NMR spectrum. 1
Figure S2: ^{13}C NMR spectrum. 2
Figure S3: ^{31}P NMR spectrum. 3
Figure S4: ^{1}H – ^{1}H COSY spectrum. 4
Figure S5: HSQC spectrum. 5

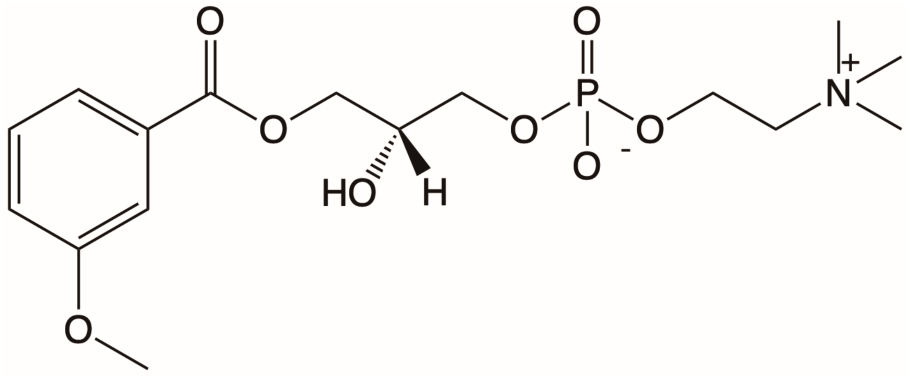

1-(3-methoxy)benzoyl-2-hydroxy-*sn*-glycero-3-phosphocholine

Figure 1. ^{1}H NMR spectrum.

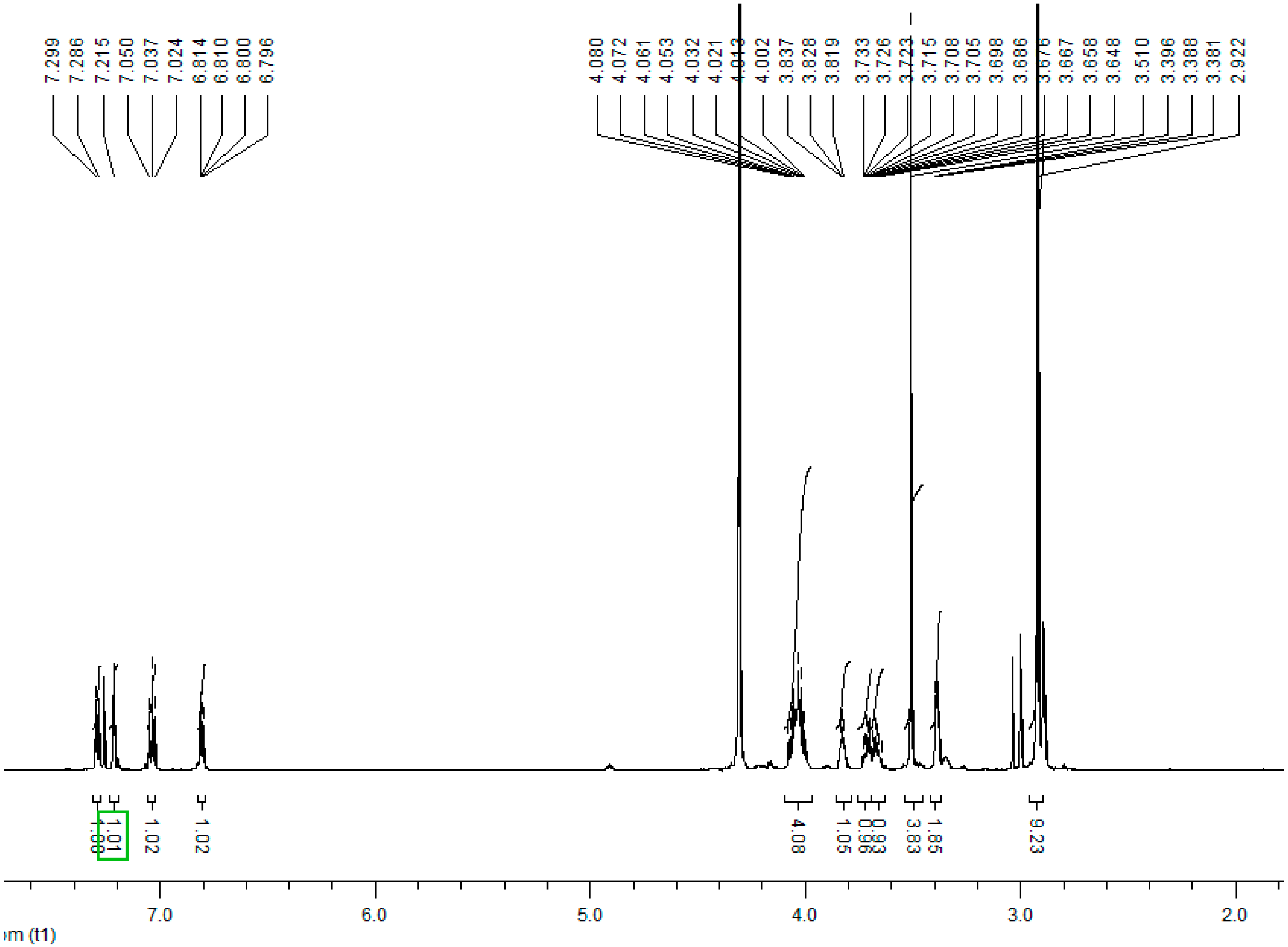

Figure 2. ^{13}C NMR spectrum.

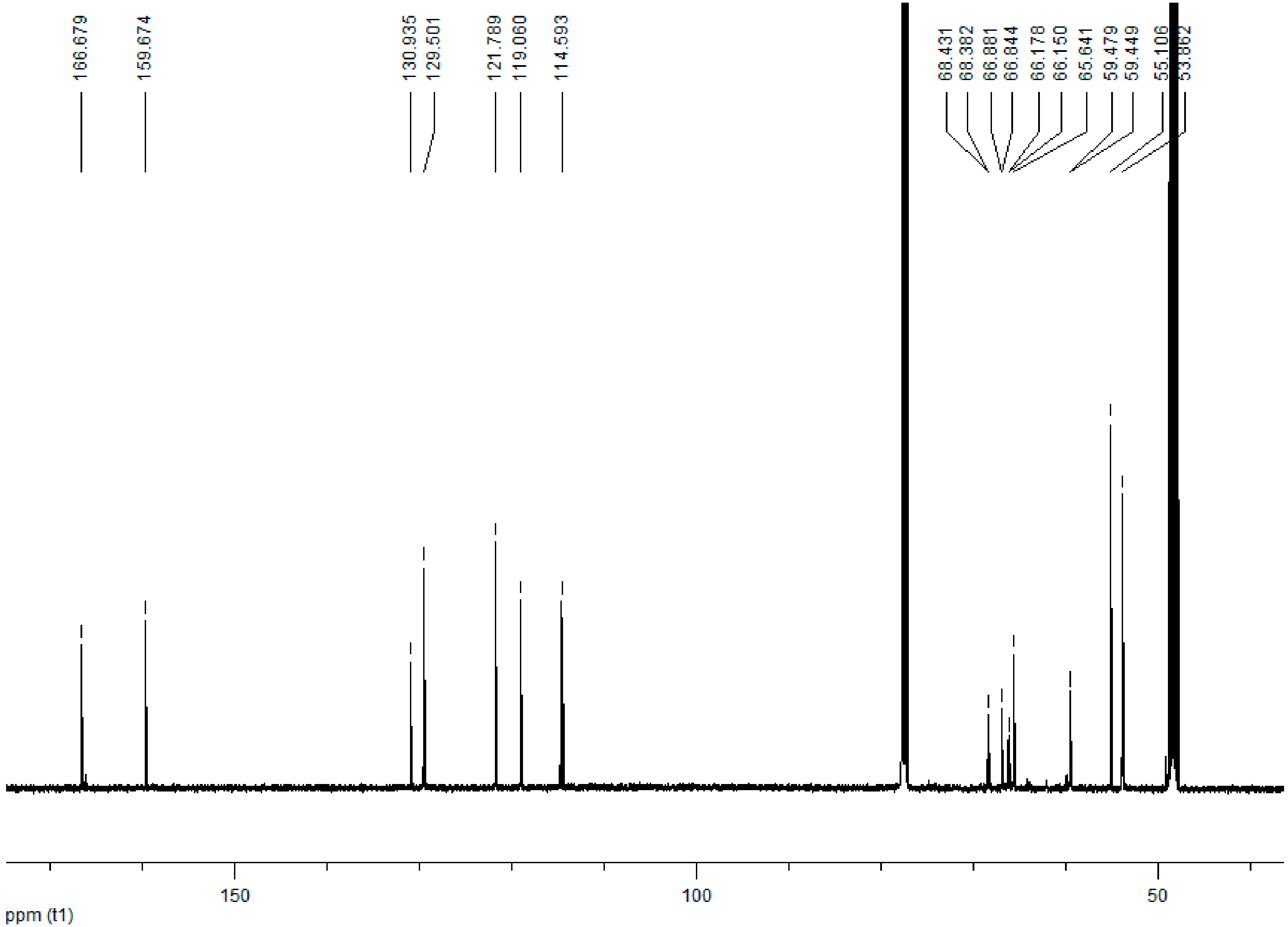

Figure 3. ^{31}P NMR spectrum.

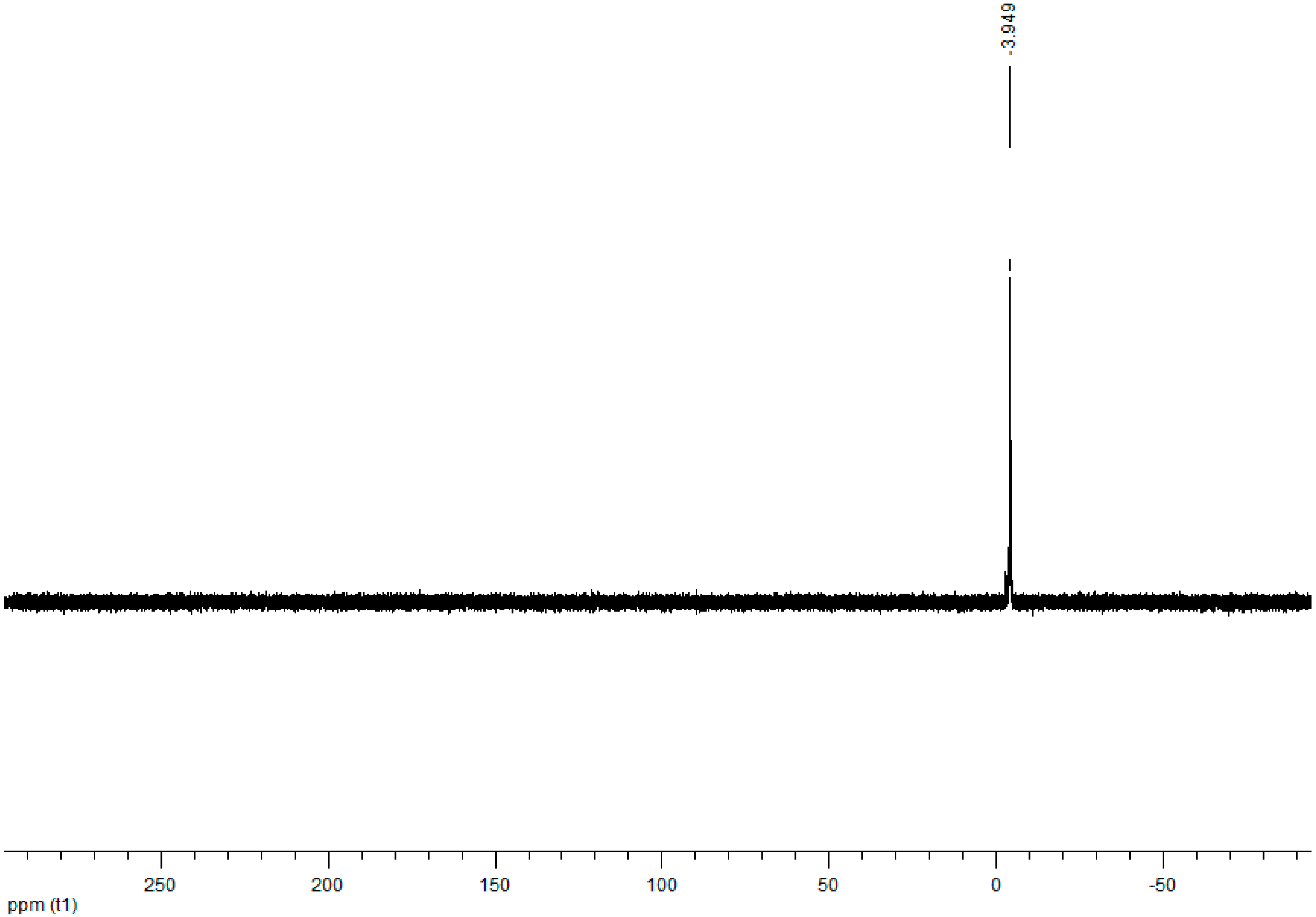

Figure 4. $^{1}H - ^{1}H$ COSY spectrum.

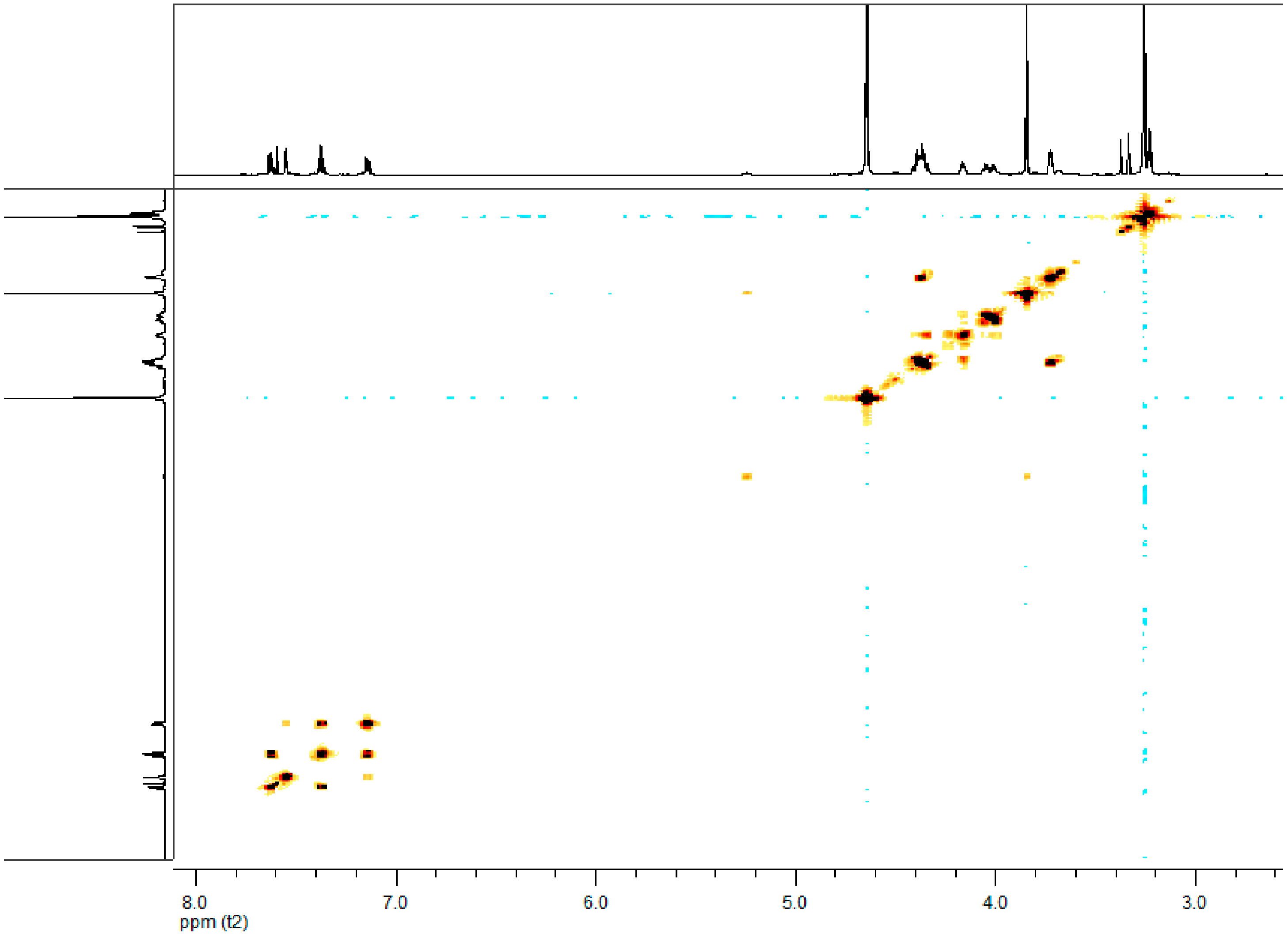

Figure 5. HSQC spectrum.

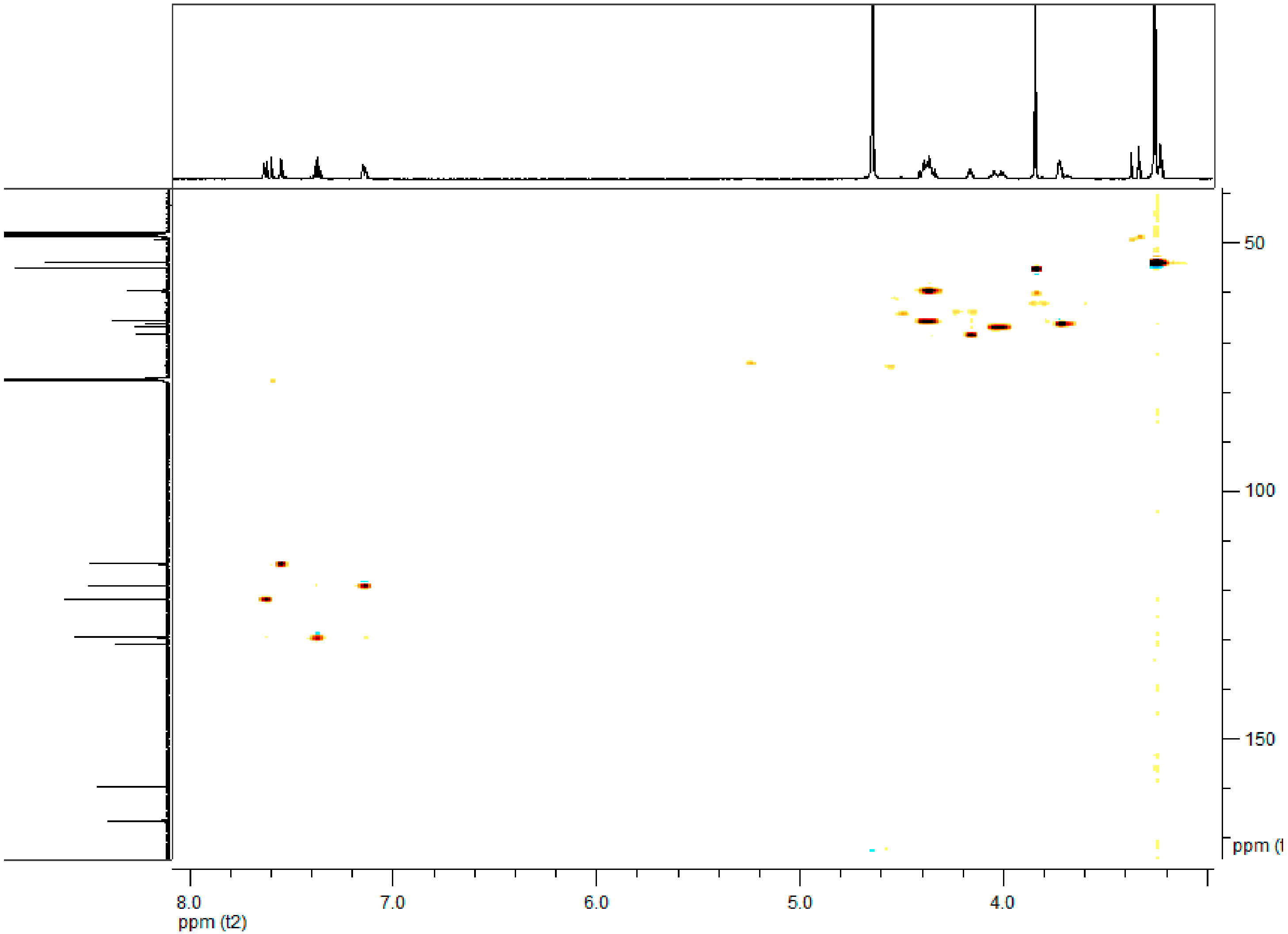

8.0
7.0
6.0
5.0
4.0
ppm (t2)
50
100
150
ppm (t1
